# Association between pathological stages and grades of chorioamnionitis and neonatal myocardial injury: a nested case–control study within a retrospective cohort

**DOI:** 10.3389/fmed.2026.1850648

**Published:** 2026-07-22

**Authors:** Min Xu, Chuanhong Wu, Jinmei Shen, Mingji Yi, Zhaochuan Yang, Liyan Wang, Chenggang Mao, Zipu Li

**Affiliations:** 1 Heart Center, Qingdao Women and Children’s Hospital, Shandong University, Qingdao, China; 2 Department of Pediatrics, The Affiliated Hospital of Qingdao University, Qingdao, China; 3 Medical Record Management Department, Qingdao Women and Children’s Hospital, Qingdao, China

**Keywords:** chorioamnionitis, multivariable logistic regression model, neonatal myocardial injury, placental pathology, systemic inflammatory response index

## Abstract

**Background:**

Although chorioamnionitis (CA) is a known risk factor for neonatal complications, the epidemiological links between specific placental inflammatory stages, systemic biomarkers, and neonatal myocardial injury (NMI) remain unclear.

**Methods:**

This nested case–control study, derived from a retrospective cohort of 24,175 births, included 942 neonates (314 cases and 628 controls) delivered from 2022 to 2024. Standard and Firth’s penalized logistic regression analyses identified independent risk factors for NMI. Predictive performance was evaluated using receiver operating characteristic (ROC) curves, with internal validation performed via the BCa bootstrap method. Model reliability and clinical utility were further assessed using calibration plots and decision curve analysis (DCA), focusing on the optimized Combined model 2.

**Results:**

Maternal inflammatory response (MIR) and fetal inflammatory response (FIR) stages and grades were significantly higher in the case group (*p* < 0.001). Multivariable logistic regression identified MIR (stage and grade), PROM, and neonatal SIRI as independent predictors of NMI (*p* < 0.05). Notably, FIR stage demonstrated a strong and significant association with NMI in univariate analysis. MIR stage showed the highest individual predictive accuracy (AUC = 0.814). Combined model 2 exhibited superior discriminatory power (AUC = 0.870), followed by Combined model 1 (AUC = 0.849). Internal validation confirmed the stability of these models. For the optimal Combined model 2, calibration plots revealed excellent reliability with a minimal mean absolute error of 0.02, while DCA demonstrated significant clinical net benefit across a broad threshold probability range of 0.1–0.9.

**Conclusion:**

Pathological placental MIR stage and grade are potent predictors of NMI. The synergy between PROM, SIRI, and placental inflammation underscores a continuous maternal-fetal inflammatory axis potentially involved in the development of NMI. These internally evaluated multidimensional models provide a robust framework for early risk stratification and targeted intervention for high-risk neonates.

## Introduction

Chorioamnionitis (CA) causes a wide range of neonatal morbidities. Beyond the well-documented pulmonary, neurological, and retinal sequelae (1–5), its systemic repercussions, particularly those affecting the developing cardiovascular system, remain insufficiently characterized.

Emerging evidence indicates that CA-related inflammation extends beyond traditional organ involvement, disrupting cardiac programming and impairing functional integrity. The fetal heart is highly sensitive to placental function and hemodynamic fluctuations; therefore, placental injury may impair cardiac development (6–9). Beyond damage to the placental barrier, CA-associated immune shifts disturb gene networks essential for morphogenesis and angiogenesis, potentially leading to long-term structural anomalies (10, 11). *In vivo* models support these findings. Intrauterine inflammation in preterm lambs restricts cardiomyocyte maturation and induces maladaptive left ventricular remodeling. Furthermore, lipopolysaccharide exposure increases myocardial collagen deposition and immune infiltration, thereby exacerbating the effects of prematurity on myocardial growth and increasing the long-term risk of cardiovascular dysfunction (12).

Neonatal myocardial injury (NMI) is a clinical condition characterized by biochemical evidence of myocardial damage, such as elevated cardiac enzymes (e.g., CK-MB), often accompanied by subtle clinical signs of cardiac dysfunction or hemodynamic instability. Although often subclinical in its early stages, NMI can significantly compromise neonatal transition and has been linked to increased morbidity in the intensive care setting.

Fetuses with premature rupture of membranes (PROM) showed signs of cardiac remodeling and subclinical dysfunction, which were more pronounced in those exposed to CA. These findings suggest that neonatal cardiovascular abnormalities originate within the intrauterine inflammatory milieu and may persist into adulthood (13). As conventional risk factors do not fully explain adult cardiovascular disease, the current consensus increasingly attributes its origins to the fetal-neonatal transition. Specifically, perinatal inflammatory exposure, through “fetal programming,” induces durable modifications that heighten cardiovascular vulnerability throughout the lifespan (14–16).

Despite its clinical significance, most existing literature focuses on the long-term structural remodeling of the heart, while evidence that directly links CA to NMI remains limited. This gap hinders the early identification and targeted management of high-risk neonates. Therefore, clarifying CA-induced NMI is essential for improving clinical decision-making. Using a case–control design, this study investigated the impact of CA, stratified by pathological stage and grade, on NMI. This study aimed to establish a clear association between CA and NMI, providing a framework for future cardiovascular prevention strategies.

## Methods

### Study population

This nested case–control study, conducted within a retrospective, single-center cohort, was approved by the Institutional Review Board of Qingdao Women and Children’s Hospital (approval No: QFELL-YJ-2025-034). Given the retrospective design and use of de-identified data, the requirement for informed consent was waived. The study adhered strictly to the RECORD guidelines.

**Figure 1:**
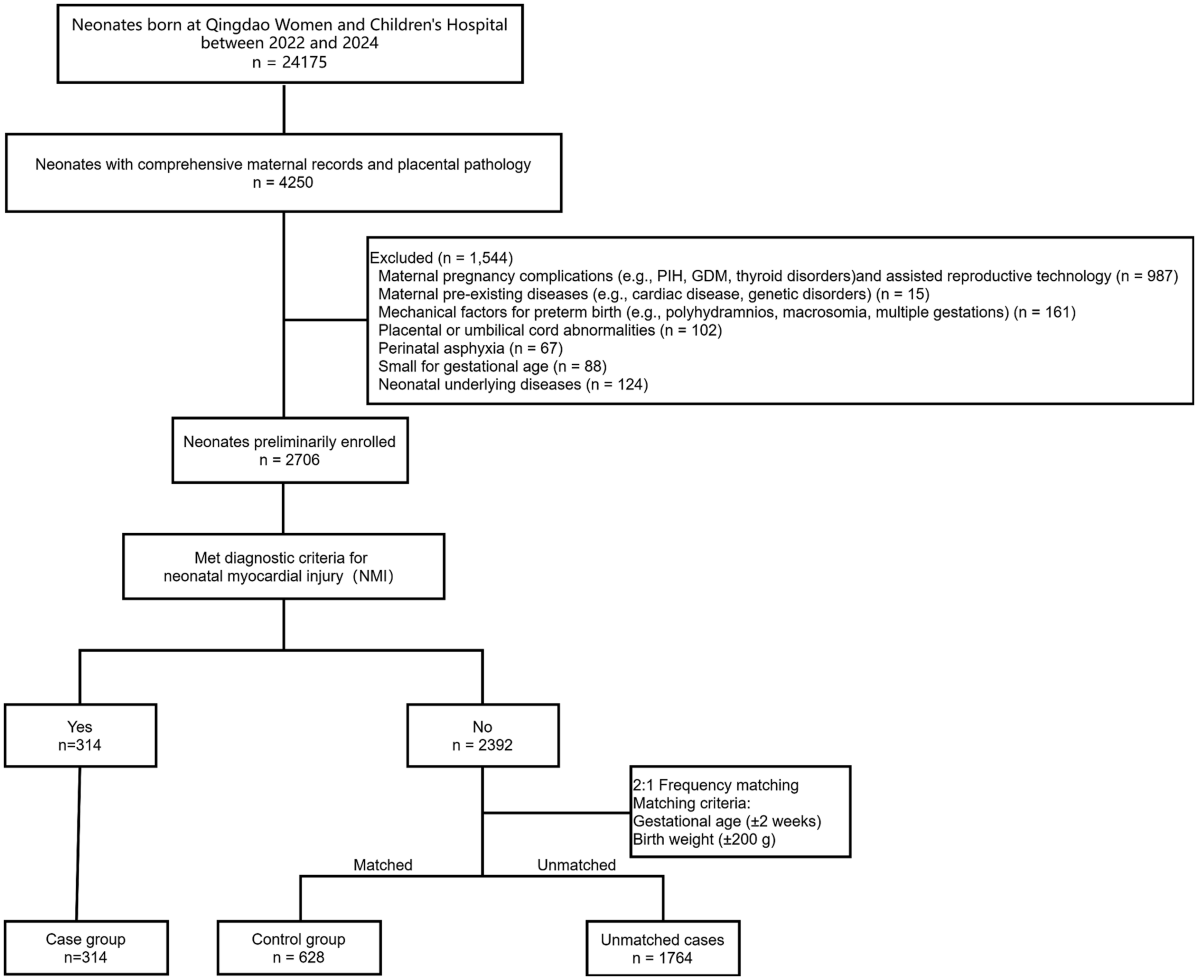
Flowchart recruitment process.

Initially, a total of 24,175 neonates born between 2022 and 2024 at Qingdao Women and Children’s Hospital were considered for inclusion. From this cohort, 4,250 neonates with comprehensive maternal records and placental pathology reports were screened. Clinical data were extracted from electronic medical records at the hospital. This specific analytic subset was intentionally selected to comprise only patients with fully available placental pathology reports and absolutely no major maternal or neonatal comorbidities, thereby isolating the independent histopathological effects. The inclusion criteria required comprehensive maternal prenatal records and definitive placental pathological findings. Patients with congenital neonatal diseases, small-for-gestational-age status, maternal comorbidities, conception via assisted reproductive technology, pregnancy-related complications, mechanical triggers for preterm birth, placental or umbilical cord anomalies, and perinatal asphyxia were excluded. Based on these criteria, a retrospective cohort of 2,706 eligible neonates were included. From this established cohort, 314 neonates who met the diagnostic criteria for NMI were identified as the case group. The matched control group (*n* = 628) was subsequently selected from the remaining 2,392 neonates using 1:2 individual frequency matching based on gestational age (±2 weeks) and birth weight (±200 g). This matching strategy was employed to ensure that the baseline clinical characteristics were balanced between the two groups, thereby minimizing the confounding effects of maturity and physical development on NMI. The final sample size was determined based on the total number of eligible cases available during the study period, providing sufficient statistical power to detect meaningful differences between the groups (Figure 1).

### Diagnostic criteria

Placental pathology was evaluated according to the Amsterdam Placental Workshop Group Consensus Statement (17). Using this standard, maternal inflammatory response (MIR) and fetal inflammatory response (FIR) were systematically categorized by stage (1–3) and grade (1–2) (Table 1). All placental slides were independently reviewed by two senior pathologists blinded to clinical outcomes. Discrepancies were resolved through consensus.

**Table 1:** Amsterdam criteria for placental pathological staging and grading of CA.

Category	MIR	FIR
Stages		
Stage 1	Acute subchorionitis or chorionitis	Chorionic vasculitis or umbilical venitis
Stage 2	Acute CA	Involvement of umbilical vein and one or more umbilical arteries
Stage 3	Necrotizing CA	Necrotizing funisitis or concentric perivasculitis
Grades		
Grade 1	Mild to moderate inflammatory changes	Mild to moderate inflammatory changes
Grade 2	Severe (confluent) inflammatory changes [≥10 neutrophils/20 × high-power field (HPF)] or subchorionic microabscesses	Severe inflammatory changes with necrosis of vascular smooth muscle

MIR, maternal inflammatory response; FIR, fetal inflammatory response; CA, chorioamnionitis.

The diagnosis of NMI was established based on an integrative framework of clinical, biochemical, and imaging findings, adapted from established clinical practices to ensure high specificity (18, 19). This multidimensional approach required at least one manifestation from criterion 1 occurring along with at least one finding from criteria 2 to 4: 1. Clinical manifestations: Including at least one of the following: (1) persistent bradycardia (heart rate < 100 bpm) or muffled heart sounds; (2) acute irritability, cyanosis, or clinical signs of congestive heart failure; (3) impaired peripheral circulation, characterized by pallor, acrocyanosis, and a capillary refill time (chest) ≥ 3 s; and (4) severe arrhythmia and/or cardiac arrest. 2. Biochemical evidence: elevated creatine kinase-MB (CK-MB) isoenzyme levels (≥ 40 U/L), measured within the first 24–48 h of life. To enhance cardio-specificity, these measurements were interpreted in conjunction with the infant’s clinical status to ensure the exclusion of potential interference from skeletal muscle trauma (18). 3. Electrocardiographic alterations: Persistent ST-T segment changes in lead II or V5 lasting > 2 days. 4. Echocardiographic evidence of myocardial dysfunction: including reduced left ventricular ejection fraction (LVEF < 55%), abnormal wall motion, tricuspid regurgitation, or diminished myocardial contractility as assessed by Doppler tissue imaging within the early postnatal period.

The maternal systemic inflammatory response index (SIRI) was used to assess inflammatory status across different gestational trimesters. The blood samples for calculating SIRI were collected at standardized clinical time points: first-trimester SIRI was measured during the initial prenatal registration (8–12 weeks of gestation); second-trimester SIRI was measured during the routine fetal anomaly scan (20–24 weeks of gestation); and third-trimester SIRI was measured upon admission for delivery. It is calculated using the following formula:


$$\text{SIRI}=\frac{\text{absolute neutrophil count}\times \text{absolute monocyte count}}{\text{absolute lymphocyte count}}$$

Neonatal infection markers were evaluated using neonatal SIRI and C-reactive protein (CRP) levels measured within the first 24 h of life.

### Statistical analysis

Data analyses were performed using SPSS (version 27.0), GraphPad Prism (version 10.1.2), and R software (version 4.3.1). The normality of continuous variables was assessed using the Kolmogorov–Smirnov test. Normally distributed data are expressed as mean ± standard deviation (SD) and compared using the independent samples *t*-test. When variances were unequal, the Welch’s *t’*-test was applied. Non-normally distributed variables were presented as medians with interquartile ranges (*M* [*Q1*–*Q3*]) and were compared using the Mann–Whitney *U* test. Categorical variables, expressed as frequencies and percentages, were compared using the *χ*^2^ test, Yates’ continuity correction, or Fisher’s exact test, as appropriate.

Univariate logistic regression analysis was conducted to screen for potential risk factors. To address potential bias from sparse data and quasi-complete separation in certain placental pathology subgroups, Firth’s penalized likelihood logistic regression was performed using the logistf package in R software. This method provides more robust estimates than standard maximum likelihood estimation when event rates are extremely high or low in subcategories.

Independent risk factors for NMI identified from the multivariable analyses were utilized to construct progressive, multidimensional combined risk assessment models (Combined model 1 and Combined model 2). The predictive performance of individual factors and the integrated models were evaluated using receiver operating characteristic (ROC) curves. The area under the curve (AUC) and the corresponding 95% confidence intervals (CIs) were calculated, with internal validation performed via the BCa bootstrap method with 2,000 resamples. Optimal cut-off values were determined using the maximum Youden index. Comparative analysis of AUCs was performed using the DeLong test, with Combined model 1 serving as the reference standard. To evaluate the final optimized framework (Combined model 2), calibration curves were generated using a bootstrap method with 1,000 resamples to assess the consistency between the predicted and observed probabilities. Additionally, decision curve analysis (DCA) was conducted to assess the clinical usefulness and net benefit of this optimized model. To address the inherent biological dependency between MIR and FIR, both factors were initially included in the risk assessment model to evaluate their relative contributions. This approach allowed for the identification of MIR as the primary factor within the maternal-fetal inflammatory sequence while accounting for overlapping variance. To ensure the transparency and reproducibility of the resulting risk assessment framework, the specific regression coefficients and the formal diagnostic equation were explicitly reported in the Results section, facilitating independent verification and clinical utility assessment. Statistical significance was set at a two-tailed *p* < 0.05.

## Results

### Maternal and neonatal clinical characteristics

A total of 942 neonates were enrolled, including 314 in the case group and 628 in the control group. As shown in Table 2, there were no significant differences in gestational age and birth weight between the two groups (both *p* > 0.05), which were used as matching variables. Maternal age distribution differed significantly between the two groups (*p* = 0.001). Although maternal ages primarily ranged from 26 to 35 years in both groups, advanced maternal age (36–40 years) was more frequent in the case group (21.97% vs. 13.22%). In contrast, younger maternal age (21–25 years) was less frequent among cases (3.50% vs. 7.17%). Additionally, the mean prenatal body mass index (BMI) was significantly higher in the case group (*p* = 0.034). The delivery mode also differed significantly (*p* = 0.006), with a higher rate of cesarean sections (46.50% vs. 37.10%).

**Table 2:** Comparison of maternal and neonatal clinical characteristics.

Variables	Case group (*n* = 314)	Control group (*n* = 628)	*χ*^2^/*t*	*p*
Matching variables				
Gestational age (weeks)	36.74 ± 4.06	36.69 ± 3.79	**0.202**	**0.840**
Birth weight (g)	2,854.21 ± 851.7	2,850.64 ± 853.12	**0.061**	**0.952**
Maternal age (years)			**19.13**	**0.001**
21–25	11	45		
26–30	94	239		
31–35	133	246		
36–40	69	83		
41–45	7	15		
Prenatal BMI (kg/m^2^)	28.53 ± 3.87	27.97 ± 3.85	**2.119**	**0.034**
Gravidity	1.79 ± 1.19	1.89 ± 1.22	**1.18**	**0.238**
Parity	1.24 ± 0.49	1.28 ± 0.52	**1.194**	**0.233**
Number of abortions	0.54 ± 0.96	0.61 ± 1.01	**1.000**	**0.318**
Mode of delivery			**7.684**	**0.006**
Vaginal delivery	168	395		
Cesarean section	146	233		

Continuous variables (prenatal BMI, gravidity, parity, abortions) are shown as mean ± SD and compared by independent samples *t*-test. Categorical variables (maternal age distribution, delivery mode) are presented as frequencies and compared using the chi-square (*χ*^2^) test. Matching variables include gestational age and birth weight (1:2 frequency matching). *p* < 0.05. Bold values indicate matching variables.

### Distribution of placental MIR and FIR stages and grades

**Figure 2:**
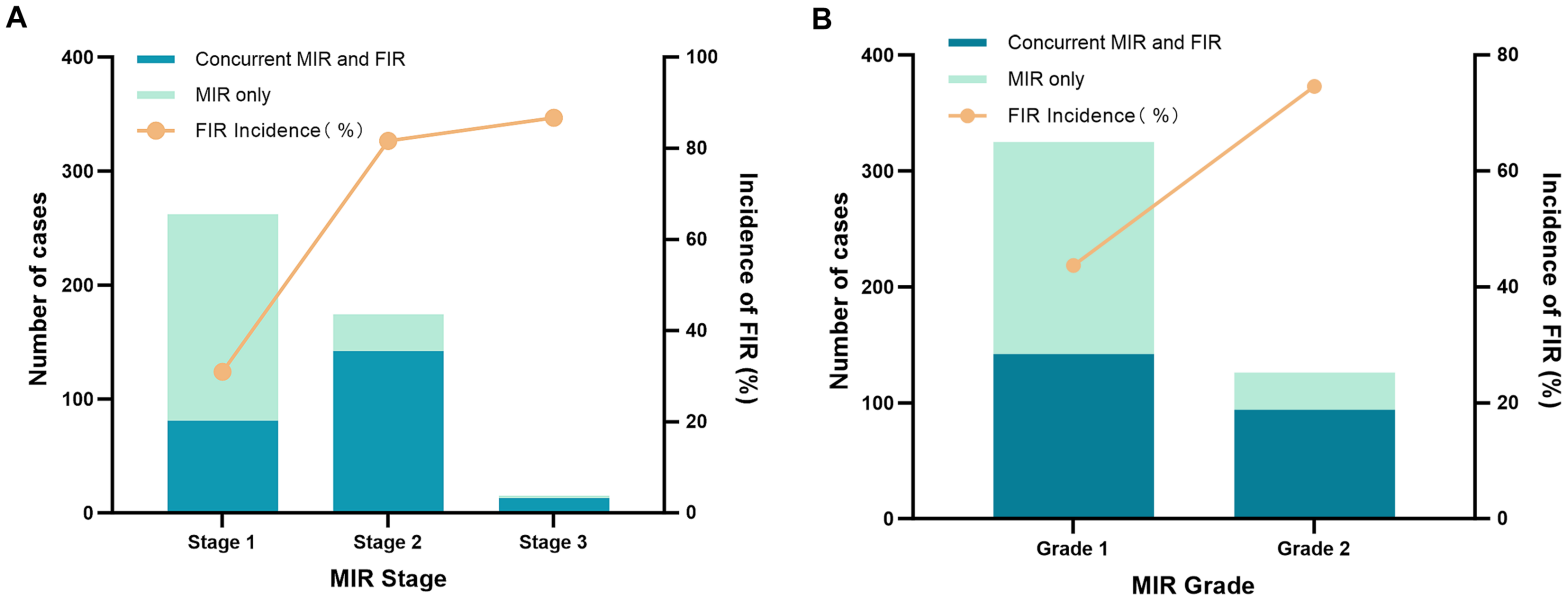
**(A)** FIR incidence by MIR stage. Bars represent MIR cases: light blue (MIR-only), dark blue (MIR with FIR). The line graph indicates increased FIR incidence with advanced MIR stages (30.92, 81.61, 86.67% for Stages 1–3; *p* < 0.001). MIR, maternal inflammatory response; FIR, fetal inflammatory response. **(B)** FIR incidence by MIR grades. Bars represent MIR cases: light blue (MIR-only), dark blue (MIR with FIR). The line graph indicates increased FIR incidence with advanced MIR grades (43.69 and 74.60% for Grades 1 and 2, respectively; p < 0.001). MIR, maternal inflammatory response; FIR, fetal inflammatory response.

Placental MIR and FIR were identified in 451 (47.88%) and 236 (25.05%) neonates, respectively (Table 3). FIR occurred only concurrently with MIR, comprising 52.33% (236/451) of MIR cases. The FIR prevalence was significantly higher with advancing MIR stage, from 30.92% (Stage 1) to 86.67% (Stage 3) (Figure 2A). Similarly, the incidence rose from 43.69% (Grade 1) to 74.60% (Grade 2) (Figure 2B).

**Table 3:** Distribution of placental MIR and FIR by stages and grades.

Variable	MIR			FIR		
	Stage 1	Stage 2	Stage 3	Stage 1	Stage 2	Stage 3
Grade 1	227	96	2	158	33	3
Grade 2	35	78	13	3	34	5

Data are presented as frequencies (*n*). Placental staging and grading followed the Amsterdam Placental Workshop Group Consensus Statement. MIR, maternal inflammatory response; FIR, fetal inflammatory response.

### Comparison of MIR and FIR stages and grades between groups

The placental MIR and FIR stages and grades differed significantly between the case and control groups (*p* < 0.001; Table 4). The case group predominantly exhibited MIR Stage 2 and Grade 1, whereas controls mostly had MIR Stage 1 and Grade 1. FIR Stage 1 and Grade 1 were most common in both groups, although FIR was significantly less frequent among controls.

**Table 4:** Comparison of MIR and FIR stages and grades.

Variable	Case group (*n* = 314)		Control group (*n* = 628)		*χ*^2^/*t*^a^	*p* ^b^
	Grade 1	Grade 2	Grade 1	Grade 2		
MIR stage					**195.019**	**<0.001**
Stage 1	58	21	169	14		
Stage 2	87	71	9	7		
Stage 3	2	11	0	2		
FIR stage					**92.711**	**<0.001**
Stage 1	138	3	20	0		
Stage 2	33	33	0	1		
Stage 3	3	5	0	0		

Data shown as numbers. Statistical significance assessed by chi-square or Fisher’s exact tests. MIR, maternal inflammatory response; FIR, fetal inflammatory response. ^a^Statistical significance was assessed using *χ*^2^ or Fisher’s exact test, as appropriate. ^b^*p* < 0.05. Bold values indicate statistical significance (*p* < 0.05).

### Comparison of PROM between groups

Maternal PROM was significantly more frequent in the case group (66.24% vs. 35.03%; *χ*^2^ = 216.3, *p* < 0.001). The proportion of cases with PROM lasting 48–168 h was notably higher in these cases (31.21% vs. 2.23%). PROM exceeding 168 h was rare in both groups (5.41% vs. 0.16%; Table 5).

**Table 5:** Comparison of PROM characteristics between case and control groups.

PROM (h)	Case group (*n* = 314)	Control group (*n* = 628)	*χ*^2^/*t*^a^	*p* ^b^
<48	93 (29.62%)	205 (32.64%)	**216.3**	**<0.001**
48–168	98 (31.21%)	14 (2.23%)		
168–672	17 (5.41%)	1 (0.16%)		

Data are presented as n (%). PROM, premature rupture of membranes. PROM duration was calculated from membrane rupture to delivery. ^a^Statistical significance was assessed using *χ*^2^ or Fisher’s exact test, as appropriate. ^b^*p* < 0.05.

### Comparison of maternal SIRI across gestational stages and neonatal inflammatory markers

Maternal SIRI differed significantly between the groups only during the third trimester (*p* < 0.001). Neonatal SIRI and CRP levels were significantly higher in the case group than in the control group (*p* < 0.001; Table 6).

**Table 6:** Maternal SIRI and neonatal inflammatory markers.

Variable	Case group (*n* = 314)	Control group (*n* = 628)	*Z* ^a^	*p* ^b^
Maternal SIRI				
First trimester	1.94 (1.33–3.00)	1.93 (1.33–2.75)	**−0.434**	**0.664**
Second trimester	2.09 (1.48–3.17)	2.21 (1.60–3.06)	**−0.954**	**0.340**
Third trimester	4.72 (2.51–8.39)	3.57 (2.13–6.10)	**−5.131**	**<0.001**
Neonatal markers				
SIRI	3.42 (1.72–5.57)	2.28 (0.83–4.01)	**−6.783**	**<0.001**
CRP (mg/L)	2.25 (0.37–11.00)	1.25 (0.28–4.88)	**−3.408**	**0.001**

Data are expressed as median (interquartile range). SIRI (systemic immune-inflammation index) = (neutrophil count × monocyte count)/lymphocyte count. ^a^Mann–Whitney *U* test was used. ^b^*p* < 0.05. Bold values indicate statistical significance (*p* < 0.05).

### Univariate and multivariable logistic regression analyses of risk factors for NMI

Univariate analysis identified multiple factors significantly associated with NMI, including maternal age, prenatal BMI, PROM duration, third-trimester maternal SIRI, neonatal SIRI, and placental MIR and FIR (stages and grades). Specifically, compared with those without inflammatory response in placental pathology, patients with MIR stage 2 (OR = 63.67, 95% CI: 36.96–116.34) and MIR grade 2 (OR = 29.19, 95% CI: 17.66–50.01) had a substantially higher risk of NMI. Notably, FIR stage 2–3 (OR = 303.24, 95% CI: 81.61–2,689.20) and FIR grade 2 (OR = 168.92, 95% CI: 44.57–1,508.46) were associated with markedly elevated risks (Table 7). After adjusting for potential bias from sparse data and quasi-complete separation using Firth’s penalized likelihood correction, these placental inflammatory parameters remained powerful predictors of NMI (*p* < 0.001). Due to the strong biological collinearity between MIR and FIR (as all FIR cases were preceded by MIR), the statistical contribution of FIR was largely subsumed by MIR in the final multivariable regression. Multivariable logistic regression analysis suggested that MIR stage, MIR grade, PROM duration, and neonatal SIRI were independently associated with the risk of NMI (*p* < 0.001). Specifically, compared with those without inflammatory response in placental pathology, patients with MIR stage 2 had a significantly higher risk of NMI, with an adjusted OR of 20.17 (95% CI: 7.22–56.34). Similarly, for MIR grade, the risk of NMI was markedly elevated in grade 1 (a OR = 27.50, 95% CI: 15.28–49.49) and grade 2 (a OR = 58.50, 95% CI: 7.24–473.05). Each additional hour of PROM increased the risk by 3.0% (OR = 1.03, 95% CI: 1.02–1.04). Similarly, each unit increase in neonatal SIRI increased the NMI risk by 16.0% (OR = 1.16, 95% CI: 1.07–1.26; Table 8).

**Table 7:** Univariate analysis of risk factors for NMI via standard and Firth’s penalized logistic regression.

Variable	*β*	S*_β_*	Wald *χ*^2^	*p* ^a^	OR (95% CI)
Maternal age			18.657	**0.001**	
26–30 vs.21–25	0.476	0.358	1.768	**0.184**	**1.61 (0.80–3.24)**
31–35 vs.21–25	0.794	0.353	5.052	**0.025**	**2.21 (1.11–4.42)**
36–40 vs.21–25	1.224	0.374	10.727	**0.001**	**3.40 (1.64–7.08)**
41–45 vs.21–25	0.647	0.568	1.296	**0.255**	**1.91 (0.63–5.81)**
Prenatal BMI	0.038	0.018	4.456	**0.035**	**1.04 (1.00–1.08)**
PROM	0.036	0.003	113.150	**<0.001**	**1.04 (1.03–1.04)**
MIR stage (ref: none)			377.441	**<0.001**	
Stage 1	1.055	0.189	31.212	**<0.001**	**2.87 (1.98–4.17)**
Stage 2	4.154	0.291	202.066	**<0.001**	**63.67 (36.96–116.34)**
Stage 3	3.578	0.701	39.546	**<0.001**	**35.79 (10.54–185.29)**
MIR grade (ref: none)			250.998	**<0.001**	
Grade 1	1.701	0.174	250.998	**<0.001**	**5.48 (3.91–7.74)**
Grade 2	3.374	0.268	250.998	**<0.001**	**29.19 (17.66–50.01)**
FIR stage (ref: none)			491.412	**<0.001**	
Stage 1	3.741	0.260	491.412	**<0.001**	**42.14 (25.86–71.93)**
Stage 2–3	5.715	0.832	491.412	**<0.001**	**303.24 (81.61–2,689.20)**
FIR grade (ref: none)			485.066	**<0.001**	
Grade 1	3.951	0.257	485.066	**<0.001**	**51.97 (32.08–88.32)**
Grade 2	5.129	0.838	485.066	**<0.001**	**168.92 (44.57–1,508.46)**
Third-trimester SIRI	0.054	0.013	18.905	**<0.001**	**1.06 (1.03–1.08)**
Neonatal SIRI	0.171	0.028	37.853	**<0.001**	**1.19 (1.12–1.25)**

NMI, neonatal myocardial injury (dependent variable: 0 = no, 1 = yes); ref: none, indicates no inflammatory response in placental pathology; MIR, maternal inflammatory response; FIR, fetal inflammatory response; SIRI, systemic immune-inflammation index; PROM, premature rupture of membranes; OR, odds ratio; CI, confidence interval. For placental pathological parameters (MIR and FIR), Firth’s penalized likelihood logistic regression was applied to address sparse-data bias and quasi-complete separation. *p* values and *χ*^2^ values for these parameters were derived from penalized likelihood ratio tests to ensure statistical robustness.

^a^*p* < 0.05. Bold values indicate statistical significance (*p* < 0.05).

**Table 8:** Multivariable logistic regression analysis for independent NMI risk factors.

Variable	*β*	S*_β_*	Wald*χ*^2^	*p* ^a^	a OR (95% CI)
MIR stage (ref: none)			39.214	<0.001	
Stage 1	0.773	0.487	2.523	0.112	2.17 (0.84–5.63)
Stage 2	3.004	0.524	32.875	<0.001	20.17 (7.22–56.34)
Stage 3	1.310	1.085	1.457	0.227	3.71 (0.44–31.07)
MIR grade (ref: none)			129.903	<0.001	
Grade 1	3.314	0.300	122.220	<0.001	27.50 (15.28–49.49)
Grade 2	4.069	1.066	14.560	<0.001	58.50 (7.24–473.05)
PROM	0.029	0.005	42.033	<0.001	1.03 (1.02–1.04)
Neonatal SIRI	0.152	0.042	13.310	<0.001	1.16 (1.07–1.26)

NMI, neonatal myocardial injury (dependent variable: 0 = no, 1 = yes); ref: none, indicates no inflammatory response in placental pathology; a OR, adjusted odds ratio; CI, confidence interval; MIR, maternal inflammatory response; PROM, premature rupture of membranes; SIRI, systemic immune-inflammation index. A multivariable logistic regression model was constructed using a forward stepwise (Likelihood Ratio) method. ^a^*p* < 0.05.

Multivariable logistic regression analysis was structured into two separate evaluation pathways to avoid over-fitting and to distinctively assess the impacts of placental inflammatory topography versus severity. Model 1 integrated maternal clinical characteristics and placental anatomical progression (MIR stages), whereas Model 2 incorporated clinical characteristics and histological severity (MIR grades). Based on the parameters estimated in Table 8, the risk assessment equations for each model are established as follows:

Model 1 (Stage-based assessment equation):


$$\begin{array}{c} \text{logit}(P_{1})=-12.569+0.773\times \mathrm{MIR}\;\text{stage}\;1+3.004\times \mathrm{MIR}\;\text{stage}\;2\\ +1.310\times \mathrm{MIR}\;\text{stage}\;3+0.029\times \text{PROM}\\ +0.152\times \text{Neonatal SIRI} \end{array}$$

Model 2 (Grade-based assessment equation):


$$\begin{array}{c} \text{logit}(P_{2})=-12.569+3.314\times \mathrm{MIR}\;\text{grade}\;1+4.069\times \mathrm{MIR}\;\text{grade}\;2\\ +0.029\times \text{PROM}+0.152\times \text{Neonatal SIRI} \end{array}$$

Where *P*_1_ and *P*_2_ represent the predicted probability of neonatal myocardial injury (NMI) calculated by the respective models. These equations provide a transparent, reproducible, and internally evaluated framework for individual risk assessment, allowing clinicians to quantitatively evaluate the likelihood of NMI based on specific placental inflammatory configurations and systemic biomarkers.

### Predictive value of risk factors for NMI

**Figure 3:**
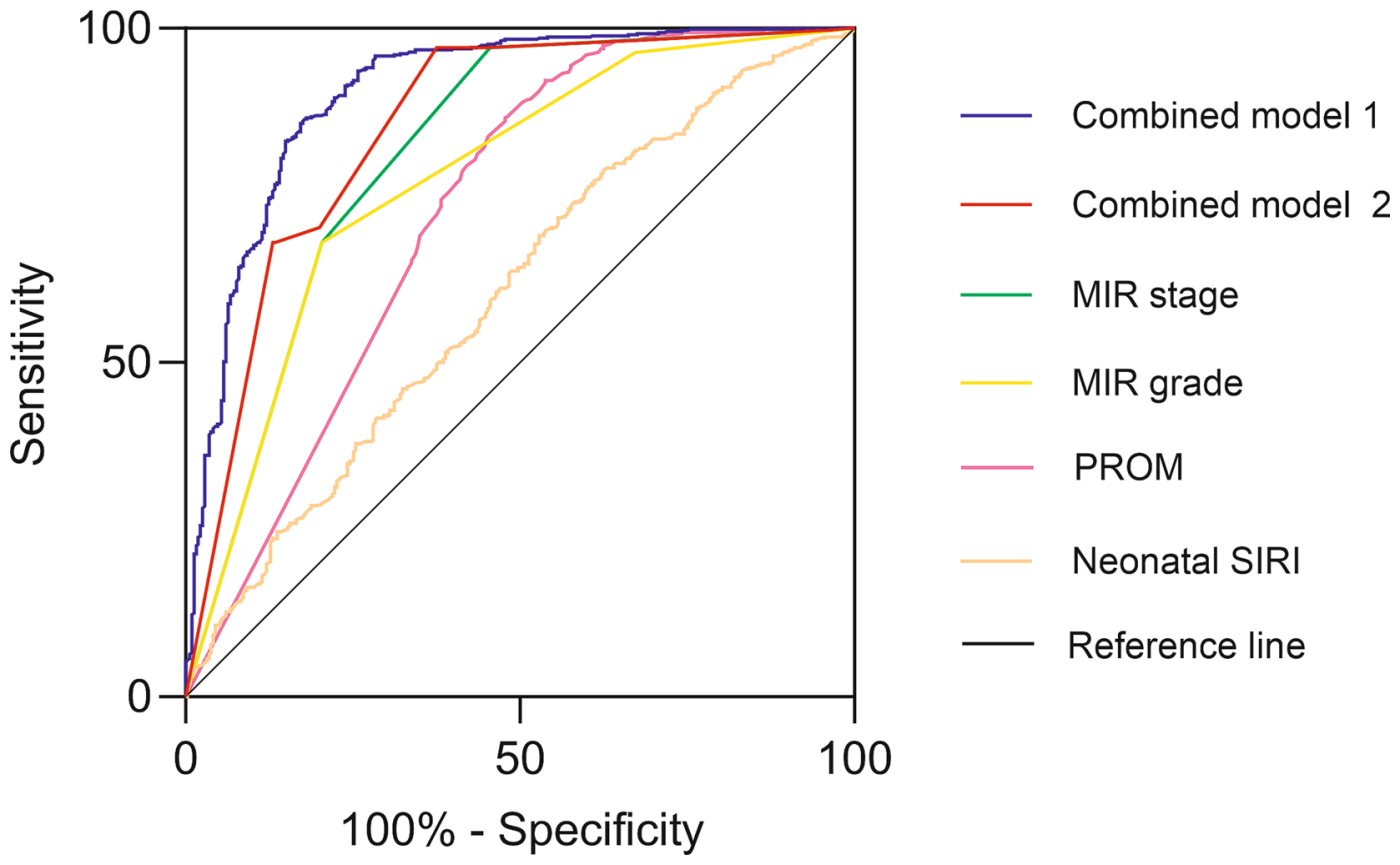
ROC curve analysis of risk factors and combined models for NMI. Receiver operating characteristic (ROC) curves illustrate predictive performance for individual risk factors and two combined models. The blue and red lines represent Combined model 1 and Combined model 2, respectively. The black diagonal line indicates reference performance (AUC = 0.5). AUC, area under the curve; MIR, maternal inflammatory response; PROM, premature rupture of membranes; SIRI, systemic inflammation response index; NMI, neonatal myocardial injury.

**Figure 4:**
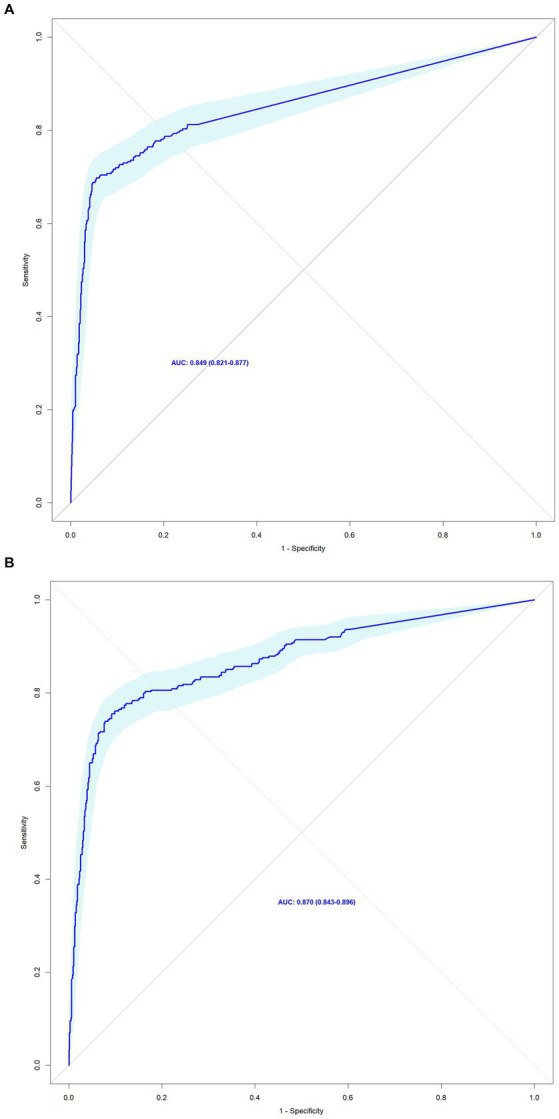
Internal validation of the combined models for predicting NMI. The plots illustrate the Receiver Operating Characteristic (ROC) curves validated through 2000 bootstrap iterations. **(A)** Combined Model 1 with an Area Under the Curve (AUC) of 0.849 (95% CI: 0.821–0.877); **(B)** Combined Model 2 with an AUC of 0.870 (95% CI: 0.843–0.896). The solid blue lines represent the mean ROC curves, and the light blue shaded areas denote the 95% confidence intervals of the AUC. NMI, neonatal myocardial injury.

ROC curve analysis was performed to evaluate the predictive performance of inflammatory markers for NMI (Figure 3). Among individual parameters, MIR stage showed the strongest individual discriminatory power with an AUC of 0.814 (95% CI: 0.776–0.849). Combined risk assessment models outperformed single variables, with Combined model 2 having the highest discrimination (AUC = 0.870, 95% CI: 0.843–0.896) and a sensitivity of 76.1%. Combined model 1, although lower in AUC (0.849, 95% CI: 0.821–0.877), demonstrated higher specificity (94.4%), which is ideal for minimizing false positives (Table 9; Figure 4). Furthermore, the DeLong test confirmed that the AUC of Combined model 2 was significantly higher than that of Combined model 1 (*p* = 0.022).

**Table 9:** Predictive performance and internal validation of combined models and clinical parameters for NMI.

Predictors	AUC	95% CI (Bootstrap)^a^	Sensitivity	Specificity	Youden index	Optimal cut-off ^b^	*p* value^c^
MIR stage	0.814	0.776–0.849	0.545	0.971	0.516	>1	<0.001
MIR grade	0.776	0.732–0.816	0.796	0.680	0.476	>0	<0.001
PROM	0.726	0.689–0.759	0.522	0.866	0.389	>22	<0.001
Neonatal SIRI	0.602	0.560–0.640	0.389	0.774	0.162	>6.3	<0.001
Combined model 1	**0.849**	**0.821–0.877**	**0.697**	**0.944**	**0.642**	**>0.333**	**–**
Combined model 2	**0.870**	**0.843–0.896**	**0.761**	**0.901**	**0.662**	**>0.272**	**0.022**

^a^Internal validation was performed using the BCa bootstrap method with 2,000 iterations to calculate the 95% CI. ^b^Optimal cut-off value was determined by the maximum Youden index (*J* = Sensitivity + Specificity − 1). ^c^*p* values were derived from the DeLong test, comparing the AUC of each parameter with that of Combined model 1. NMI, neonatal myocardial injury; MIR, maternal inflammatory response; PROM, premature rupture of membranes; SIRI, systemic inflammation response index; BCa, bias-corrected and accelerated; AUC, area under the ROC curve; CI, confidence interval. Bold values indicate combined models (Combined model 1 and Combined model 2).

### Calibration of the risk assessment model

**Figure 5:**
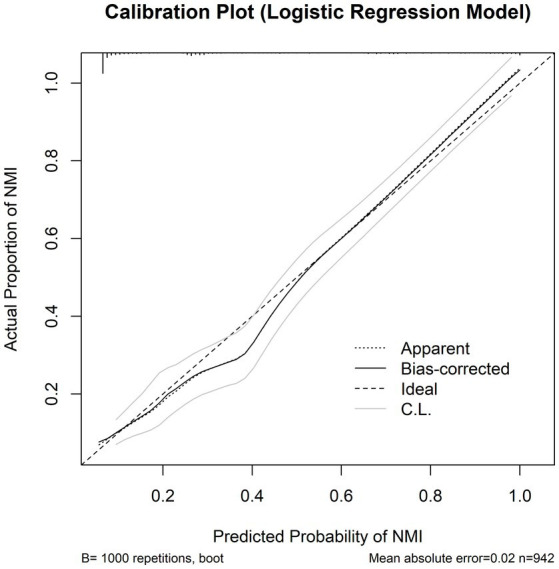
Calibration plot for the multivariable logistic regression model. The plot displays the performance of the model in predicting the probability of NMI. The *x*-axis represents the predicted probability of NMI from the model, and the *y*-axis represents the actual observed proportion of NMI. The dashed line serves as the diagonal 45-degree reference line for an ideal mode, while the solid line represents the apparent performance and the bias-corrected curve derived from 1,000 bootstrap repetitions. NMI, neonatal myocardial injury.

The calibration performance of the multivariable logistic regression model (Combined model 2) was evaluated using a calibration plot (Figure 5). The plot presents the apparent curve, the bias-corrected curve derived from 1,000 bootstrap repetitions, and the 95% confidence intervals (C.L.). The bias-corrected calibration curve demonstrated a high degree of consistency between the predicted probability of NMI and the actual observed proportion. The calibration line closely followed the 45-degree ideal reference line, as confirmed by a remarkably low mean absolute error of 0.02, indicating that the model possesses excellent calibration accuracy and reliability. There was no significant evidence of overestimation or underestimation of the NMI risk across the entire range of predicted probabilities.

### Clinical utility of the risk assessment model

**Figure 6:**
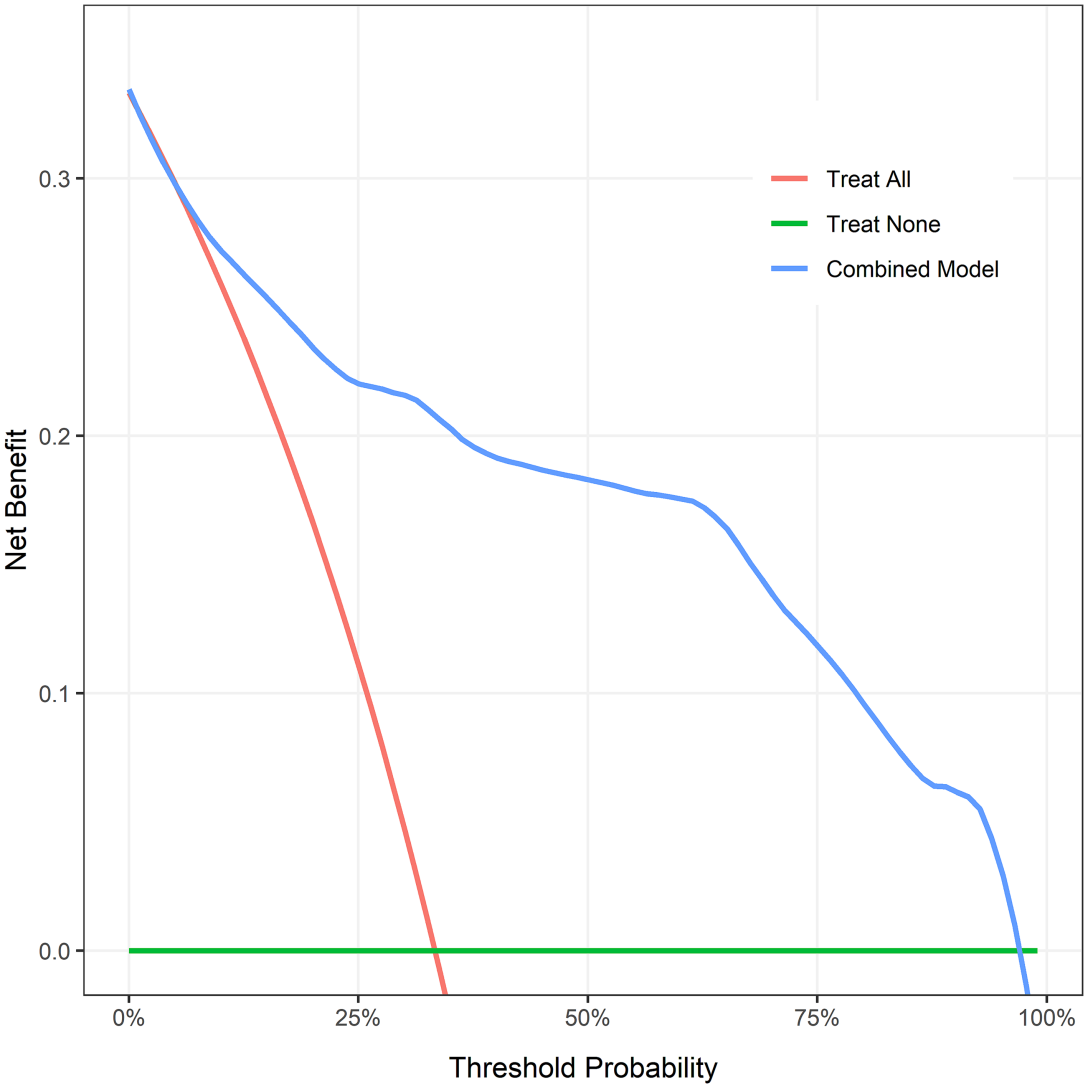
DCA for the clinical utility of the risk assessment model. The *x*-axis represents the threshold probability, and the *y*-axis represents the net benefit. The solid blue line represents the optimized Multivariable Logistic Regression framework (Combined model 2), while the solid red line (Treat All) and green horizontal line (Treat None) represent the respective clinical reference strategies. DCA, decision curve analysis; NMI, neonatal myocardial injury.

To evaluate the clinical utility and net benefit of the risk assessment model, DCA was performed (Figure 6). The blue line represents the decision curve based on Combined model 2, while the red line (Treat All) and green horizontal line (Treat None) represent the reference strategies. Within the threshold probability range of 0.1–0.9, the net benefit of using the model to identify and intervene for NMI was substantially higher than that of both reference strategies. The wide range of threshold probability indicates that regardless of clinical preferences for aggressive or conservative management, the model consistently provides significant clinical utility and has the potential to facilitate better patient outcomes without increasing unwarranted harm.

## Discussion

In this study, a 1:2 frequency matching strategy was employed. This approach not only effectively controlled for the potential confounding effects of gestational age and birth weight—two critical factors influencing neonatal cardiac function—but also optimized the statistical power without introducing excessive noise, as seen in higher-ratio matching.

Due to the subtle clinical presentation of CA and limitations in current diagnostic criteria (20), placental histopathology remains the gold standard (21). In this study, placental pathology was assessed using the Amsterdam consensus criteria, which reflect the duration and intensity of intrauterine inflammation through MIR and FIR staging and grading. This single-center, nested case–control study examined the association between these pathological metrics and NMI. The findings demonstrated that MIR and FIR stages and grades are not only diagnostic benchmarks but also factors closely associated with NMI development.

Regarding the diagnostic criteria for NMI, it must be noted that a globally unified consensus is currently lacking. In this study, we employed an integrative framework that combined biochemical biomarkers with objective clinical manifestations. While some recent literature emphasizes the high cardiac specificity of troponins, CK-MB remains a robust and widely utilized indicator for capturing early myocardial stress in neonatal settings (18, 19). By requiring the concurrence of significant enzymatic elevation with overt clinical symptoms—such as hemodynamic instability or respiratory distress—we ensured that the identified cases reflect clinically significant myocardial involvement rather than transient, subclinical fluctuations. This dual-faceted approach aligns with real-world clinical practice, ensuring that the diagnosis carries substantial prognostic weight while minimizing potential misclassification bias. Our results showed that MIR detection significantly exceeded FIR detection, with FIR occurring exclusively in cases of existing MIR. This observation supports the biological sequence hypothesis, suggesting maternal inflammation precedes fetal immune activation, with FIR initiation dependent on MIR presence (22). Further analysis revealed a stepwise increase in FIR incidence with advancing MIR stages, peaking at 86.7% at stage 3. These findings indicate that maternal inflammation progression is strongly associated with fetal inflammatory responses, potentially being linked to increased neonatal susceptibility to adverse outcomes.

### Sequential association and predictive performance of the maternal-fetal inflammatory cascade in NMI

In the case group, MIR and FIR stages and grades were significantly higher than those in the control group (*p* < 0.001). Univariate analysis identified advanced FIR as a major risk factor (stage 2–3: OR = 303.24; grade 2: OR = 168.92). This association suggested that fetal systemic inflammation is closely associated with NMI (23). The high OR for FIR stage 2–3 stems from the stage 3 subgroup, where all neonates presented with NMI. This total incidence, combined with the small sample size, results in a statistical “quasi-complete separation.” This represents a “ceiling effect,” where NMI becomes nearly certain at the peak of the inflammatory response. In the multivariable model, MIR (stage and grade), PROM duration, and neonatal SIRI remained independently associated factors. The statistical “masking” of FIR does not preclude its pathological involvement but reflects the collinearity and biological progression from MIR to FIR. As FIR constitutes the advanced phase of the maternal-fetal inflammatory cascade, its predictive variance is subsumed by MIR during simultaneous modeling.

MIR is recognized as both an initiating and sustaining factor in intrauterine inflammatory injuries (24). It represents pathogen invasion of fetal membranes and the onset of maternal pro-inflammatory cytokine influx through a compromised placental barrier. In this study, MIR grade was strongly associated with NMI, with Grade 2 conferring a 58.50-fold increased risk (a OR = 58.50, 95% CI: 7.24–473.05). The MIR stage demonstrated the highest predictive accuracy (AUC = 0.814), indicating prolonged exposure and an earlier detection window. While our cross-sectional data do not directly demonstrate a temporal causal sequence, the superior explanatory power of MIR in the regression model is consistent with its potential as an early indicator of intrauterine inflammation. Before complete fetal immune activation, MIR-induced oxidative stress, mitochondrial dysfunction, and placental hypoperfusion profoundly modulate the local immune microenvironment and fetal myocardial development (25). Thus, MIR, as the initial inflammatory event, serves as a sensitive, early, and critical indicator for evaluating the risk of neonatal cardiovascular outcomes.

Secondly, the statistical masking of FIR within the regression model reflects the sequential nature and developmental dependency of the maternal-fetal inflammatory response. This can be explained from two perspectives. First, biological collinearity: the induction of FIR is strongly contingent upon sustained stimulation from MIR (all FIR cases in this cohort had MIR). This collinearity provides MIR with superior explanatory power, overshadowing FIR, which functions as an intermediate factor. This statistical masking is consistent with the sequential nature of the inflammatory response; as advanced FIR stages occur exclusively in the presence of MIR, their shared pathological influence is predominantly captured by MIR in the multivariate analysis. Our findings thus emphasize the role of MIR as an earlier and more dominant indicator in the progression of intrauterine inflammation. Second, temporal latency and detection thresholds: the morphological manifestation of FIR in the placenta requires a distinct duration and is associated with fetal immune maturity (26).

Notably, although FIR was excluded from the final multivariate model, its exceptionally high OR value (stage 2–3: 303.24) in the univariate analysis remained significant. These findings suggest that when maternal inflammation extends to the placental barrier and aligns with fetal immune activation, the adverse effects on the neonatal myocardium may accelerate. Advanced FIR represents a critical transition in the development of NMI; however, advanced FIR may be a marker of severity of inflammatory response (disease severity) rather than an independently validated risk factor. Clinically, while MIR stages and grades serve as early indicators for risk stratification, histopathological confirmation of FIR is indicative of the progression from maternal inflammatory exposure to active fetal systemic involvement, highlighting the potential value of closer clinical surveillance in these high-risk cases.

### Synergistic effects of the maternal-fetal inflammatory cascade and SIRI

As a composite inflammatory marker reflecting systemic immune activation, SIRI demonstrates significant prognostic value in cardiovascular diseases (27–29). Elevated SIRI is also associated with adverse pregnancy outcomes, suggesting its role as a robust marker for inflammatory risks in pregnancy (30, 31). Our study identified maternal third-trimester SIRI and neonatal SIRI as independent factors associated with NMI. While the primary window for cardiac organogenesis occurs during the first trimester, the lack of SIRI elevation in early pregnancy in our cohort suggests that NMI in this context is likely an acquired inflammatory injury rather than a developmental malformation. The significant divergence of maternal SIRI specifically in the third trimester aligns with the timing of late-gestational placental barrier compromise and acute pathogen invasion. This elevation reflects extensive neutrophil infiltration into the decidua and chorion, triggering a maternal-to-fetal pro-inflammatory surge shortly before delivery. The independent association of neonatal SIRI (OR 1.16) suggests a postnatal inflammatory cascade “carry-over effect,” potentially contributing to persistent myocardial microvascular damage (32). In contrast, CRP lacked independent association with NMI risk. By integrating neutrophil, monocyte, and lymphocyte fluctuations, SIRI more precisely captures inflammatory activity, offering superior sensitivity for evaluating NMI risk.

### PROM duration: dual insult of barrier loss and mechanical compression

Multivariable regression identified PROM as an independent risk factor for NMI (adjusted OR = 1.030 per hour), highlighting the cumulative risk with increasing membrane rupture duration. Mechanistically, PROM facilitates microbial invasion, triggering a Toll-like receptor-mediated cytokine cascade, mitochondrial dysfunction, and oxidative stress in fetal cardiomyocytes (33). Concurrent oligohydramnios further raises inflammatory mediator levels and impairs fetal microcirculation through elevated intrauterine pressure, creating a harmful “ischemia-inflammation-oxidative stress” cycle (34). Although its AUC (0.726) was lower than histopathological indicators, the high sensitivity (97.1%) and easy clinical assessment of PROM duration make it a crucial indicator for NMI risk stratification.

### Weighting comparison between maternal demographic characteristics and inflammatory factors

Although the univariate analysis indicated a higher prevalence of advanced maternal age and obesity in the case group, neither factor remained independently associated with NMI after multivariate adjustment. This suggests that while advanced age may predispose to mitochondrial dysfunction and obesity may promote chronic inflammation (35–37), their contributions are overshadowed by inflammatory responses in the presence of CA. Therefore, clinical focus should prioritize dynamic inflammatory monitoring rather than baseline demographic characteristics.

### Diagnostic performance and clinical utility of the multidimensional models

The ROC analysis demonstrated high diagnostic efficacy for each parameter, where Combined model 2 exhibited the strongest discriminatory power (AUC = 0.870), followed by Combined model 1 (AUC = 0.849). Compared to individual factors, the integrated models offered superior clinical performance, with Combined model 2 achieving a balanced sensitivity and specificity (76.1 and 90.1%, respectively). To ensure the robustness of these findings, internal validation via the BCa bootstrap method confirmed the stability of these multidimensional models. Furthermore, focused evaluation on the optimal Combined model 2 was conducted; its final calibration plot revealed high agreement between estimated NMI probabilities and actual observations, as robustly quantified by a minimal mean absolute error of 0.02. Concurrently, DCA demonstrated that Combined model 2 provided significant net benefits across a broad threshold probability range of 0.1–0.9, confirming its substantial practical utility in clinical decision-making.

In summary, this evidence chain—from anatomical barrier disruption (PROM) and localized placental inflammation (MIR) to systemic fetal involvement (FIR and SIRI)—provides a reliable framework for NMI identification. While MIR characterizes the initial evaluative breadth, FIR and SIRI reflect the progression of clinical severity. Integrating these multidimensional markers into a unified risk assessment model allows for precise risk stratification, offering a robust clinical tool for early intervention and potentially better long-term neonatal outcomes.

## Conclusion

This study demonstrates that placental histopathology-confirmed MIR and FIR are independently associated with the risk of NMI. Our findings illustrate the progressive relationship from localized placental lesions to systemic fetal inflammatory activation. Prolonged PROM and elevated neonatal SIRI are synergistically associated with myocardial injury in the context of compromised physical barriers and systemic immune responses. The integrated risk assessment models exhibited high diagnostic efficacy and stability, with Combined model 2 providing the most robust discriminatory power. These results establish a clinical framework for early NMI risk stratification and management in neonates exposed to intrauterine inflammation.

As a systematic exploration of the association between placental pathology and neonatal cardiac injury, this study has several limitations. First, the single-center retrospective design and the substantial exclusion of cases with maternal comorbidities were necessary to isolate the specific effects of intrauterine inflammation, but may introduce selection bias. Importantly, our risk assessment models were developed based on this selected population and currently lack external validation. Therefore, their clinical utility and optimal thresholds require rigorous prospective validation in multi-center cohorts before wide clinical implementation. Second, the definition of NMI currently lacks a globally standardized consensus. While we utilized a multidimensional diagnostic strategy to ensure specificity, the reliance on CK-MB—which can be influenced by non-cardiac factors—remains an objective limitation compared to high-sensitivity troponin. Third, while ROC curve analysis was utilized to assess the diagnostic performance of inflammatory indicators, we acknowledge the inherent theoretical limitations of this method in biomedical applications. Its retrospective nature regarding sensitivity/specificity, assumption of data normality, and strong dependency on baseline disease prevalence mean that the derived cut-offs and AUC values should be interpreted strictly within the context of this study population, and generalizability across dynamically shifting clinical contexts warrants cautious validation. Finally, the study lacks molecular mechanistic validation and long-term cardiovascular follow-up. Future multicenter prospective studies are warranted to refine early risk assessment and intervention strategies for perinatal inflammation-related NMI.

## Data Availability

The original contributions presented in the study are included in the article/supplementary material, further inquiries can be directed to the corresponding author.
